# Cigarette Smoking Abstinence Among Pregnant Individuals Using E-Cigarettes or Nicotine Replacement Therapy

**DOI:** 10.1001/jamanetworkopen.2023.30249

**Published:** 2023-09-12

**Authors:** Xiaozhong Wen, Minseon V. Chung, Kayla A. Liszewski, Lauren D. Todoro, Eve M. Giancarlo, Wenxin Zhang, Sara K. Berkelhamer, Maciej L. Goniewicz

**Affiliations:** 1 Division of Behavioral Medicine, Department of Pediatrics, Jacobs School of Medicine and Biomedical Sciences, State University of New York at Buffalo; 2 Department of Pediatrics, University of Washington, Seattle; 3 Department of Health Behavior, Roswell Park Comprehensive Cancer Center, Buffalo, New York

## Abstract

**Importance:**

Smoking cigarettes during pregnancy can impair maternal and child health, and pregnant individuals have increasingly used electronic cigarettes (e-cigarettes) for various reasons, including quitting smoking.

**Objective:**

To assess smoking abstinence rates among pregnant individuals who used e-cigarettes compared with those who used nicotine replacement therapy (NRT).

**Design, Setting, and Participants:**

This cohort study is a secondary data analysis of phase 8 of the US Pregnancy Risk Assessment Monitoring System, conducted between 2016 and 2020. Eligible participants included pregnant individuals who smoked combustible cigarettes within the 3 months before pregnancy and either used e-cigarettes or NRT during pregnancy. Data analysis was conducted from March 2022 to April 2023.

**Exposures:**

Combustible cigarette use within 3 months before pregnancy and use of either e-cigarettes or NRT during pregnancy.

**Main Outcomes and Measures:**

The primary outcome was the individual’s self-reported smoking abstinence status during the last 3 months of pregnancy. Weighted percentages were reported and weighted multivariable logistic regression models were used to examine the association of e-cigarette use vs NRT with smoking abstinence. A propensity score was used to control for confounding by sociodemographics, pregnancy characteristics, prepregnancy smoking intensity, depression, behavioral support, and hookah use.

**Results:**

The cohort included 1329 pregnant individuals (759 ≥25 years [60.2%]; 766 non-Hispanic White individuals [79.8%]) of whom 781 had an education level of high school or lower (61.4%), and 952 had an annual household income of $48 000 or less (81.5%). Of the 1329 individuals, 890 (unweighted percentage, 67.0%) were existing e-cigarette users, 67 (unweighted percentage, 5.0%) were new e-cigarette users, and 372 (unweighted percentage, 28.0%) were NRT users. Compared with individuals who used NRT during pregnancy, individuals who used e-cigarettes had a higher rate of smoking abstinence in late pregnancy (456 individuals [50.8%] vs 67 individuals [19.4%]; propensity score adjusted odds ratio [OR], 2.47; 95% CI, 1.17-5.20; *P* = .02). In the secondary analysis stratified by the timing of e-cigarette use initiation, existing users of e-cigarettes who initiated before pregnancy had a higher smoking abstinence rate than NRT users (446 users [53.1%] vs 67 users [19.4%]; adjusted OR, 2.61; 95% CI, 1.23-5.51; *P* = .01). However, new e-cigarette users who initiated use during pregnancy had a similar smoking abstinence rate in late pregnancy when compared with NRT users (10 users [20.6%] vs 67 users [19.4%]; adjusted OR, 1.13; 95% CI, 0.22-5.87; *P* = .88).

**Conclusions and Relevance:**

These findings suggest that individuals who used e-cigarettes during pregnancy had a higher smoking abstinence rate in late pregnancy than individuals who used NRT, especially for those who initiated e-cigarette use before pregnancy, indicating that replacement of cigarettes with e-cigarettes during pregnancy may be a viable strategy for harm reduction.

## Key Points

**Question:**

What is the association of electronic cigarette (e-cigarette) use vs nicotine replacement therapy (NRT) with smoking abstinence among US pregnant individuals who smoke combustible cigarettes?

**Findings:**

In this cohort study of 1329 pregnant individuals in the US who smoked cigarettes before pregnancy, use of e-cigarettes during pregnancy was associated with a higher rate of cigarette smoking abstinence in late pregnancy compared with use of NRT. Stratified by the timing of e-cigarette use initiation, the smoking abstinence rate was higher among individuals who initiated e-cigarette use before pregnancy than NRT users, but individuals who initiated e-cigarette use during pregnancy had a similar smoking abstinence rate to NRT users.

**Meaning:**

These findings suggest that e-cigarettes have the potential to be a smoking cessation aid for pregnant individuals, especially if they initiate their use before pregnancy, indicating that replacement of cigarettes with e-cigarettes during pregnancy may also represent a strategy for harm reduction.

## Teaser

This cohort study compares cigarette smoking abstinence rates among pregnant individuals who use electronic cigarettes (e-cigarettes) vs nicotine replacement therapy (NRT).

## Introduction

Smoking cigarettes during pregnancy can severely impair both maternal and child health.^1,2^ Therefore, the American College of Obstetricians and Gynecologists (ACOG) emphasizes that smoking abstinence during pregnancy is critical for the health of both the pregnant individual and the fetus.^3^ Epidemiologic studies^4,5,6,7^ have demonstrated the benefits of maternal smoking cessation, including improved fetal growth; increased birth weight, height, and head circumference; lower risk of small-for-gestational-age birth and premature delivery; and reduced need for neonatal intensive care admission. Pregnant individuals are often motivated to quit smoking with well-publicized concerns for adverse pregnancy and birth outcomes. However, only about one-half of pregnant individuals quit smoking spontaneously, whereas many others face a variety of challenges in quitting.^8^

Although they have not yet been approved by the US Food and Drug Administration as a therapeutic product,^9^ electronic cigarettes (e-cigarettes) have been promoted as a possible smoking cessation aid given their potential for harm reduction. In support of risk reduction, gestational exposure to e-cigarettes has been associated with lower rates of small-for-gestational-age birth^10^ as well as improved neurobehavioral scoring^11^ compared with combustible cigarettes. Many known toxic chemicals present in cigarette smoke have been found at lower concentrations in e-cigarette aerosols. As an example, carbon monoxide produced with cigarette use binds to hemoglobin, causing increased hypoxia in both the mother and fetus. Prenatal exposure to carbon monoxide and hypoxia could lead to compromised blood flow and fetal growth restriction with a subsequent increase in risk of obesity, insulin resistance, and hypertension in the offspring.^12^

Although vaping can avoid numerous chemicals produced by smoking cigarettes, exposure to nicotine, flavorings, and liquid carriers remain a concern with gestational use of e-cigarettes. Animal studies^13,14,15,16^ have demonstrated alterations of endocrine, reproductive, respiratory, cardiovascular, and neurologic systems in offspring exposed to gestational nicotine alone. However, a comprehensive review^17^ of nicotine replacement therapy (NRT) in human pregnancy failed to identify any safety concerns. In vivo animal pregnancy studies evaluating the combined exposure to aerosolized nicotine, flavorings, and liquid carriers have identified impaired learning, locomotor, and memory functions in offspring.^18^ A study^19^ with rats suggested that prenatal exposure to e-cigarette vapor was associated with lower birth weight, as well as a decrease in blood flow to the maternal uterine and fetal umbilical circulation. Even e-cigarettes that are nicotine free contain aerosols and flavorings that could potentially lead to developmental toxic effects,^20^ as well as compromised lung development and pulmonary function in the fetus.^21^ For example, flavoring chemicals in e-cigarettes have been shown to impact hippocampal development with microglia activation and altered expression of neurotrophins in mice.^22^ However, there is a lack of human research to examine the potential association of e-cigarette flavorings or the constituents in e-cigarettes with outcomes in the fetus and pregnant individual.

Pioneering research has begun to compare e-cigarettes with NRT as smoking cessation methods for pregnant smokers. A randomized clinical trial (RCT)^23^ in the UK assigned smoking pregnant individuals to either refillable tobacco-flavored e-cigarettes or NRT for 8 weeks as a smoking cessation aid. To be eligible, participants could not be using e-cigarettes or NRT at the time of enrollment. Results showed that pregnant individuals randomized to receive e-cigarettes had higher self-reported quitting rates at 4 weeks (89 of 571 individuals [15.6%] in the e-cigarette group) than pregnant individuals randomized to receive NRT (61 of 569 individuals [10.7%]) (*P* = .02).^23^ However, the generalizability of these results to other countries, including the US, remains to be seen with differences in populations, public attitudes toward tobacco control policies, and NRT vs e-cigarette products. For example, an international survey^24^ showed that cigarette users in England had a higher level of support for reducing nicotine content in cigarettes, but a lower level of support for banning menthol cigarettes or other cigarette additives, compared with their counterparts in the US. In addition, popular e-cigarette products in the US often have higher nicotine content than those in EU (5.0% vs ≤1.7% in 2019).^25^ In contrast, concentrations of flavoring chemicals have been shown to be higher in EU e-cigarette products than those in the US.^25^ These differences in e-cigarette products could impact patterns of e-cigarette use and smoking abstinence rates.^25^ Currently, limited information is known about the association of e-cigarettes with smoking cessation among US pregnant individuals using these products.

In addition, the aforementioned UK trial^23^ did not include cigarette smokers who were also e-cigarette users (dual users) before pregnancy, which represents the large proportion of e-cigarette users in an empirical setting. For example, US national data^26^ collected from 2016 to 2018 showed approximately one-half of pregnant e-cigarette users were dual users. The timing of initiation of e-cigarette use may be critical in the context of using these products as a smoking cessation aid because sufficient transition time is needed for some cigarette smokers to adjust to and overcome potential challenges. Common challenges include perceived flaws of the e-cigarette technology, the taste and flavors, the sensation of vaping, and the pricing.^27^ Initiation before pregnancy provides time to experiment with products and gain experience with using e-cigarettes prior to the dramatic physiologic changes that occur during pregnancy. Therefore, it is reasonable to hypothesize that dual users may have an easier transition from dual use to exclusive e-cigarette use than those who initiated e-cigarettes during pregnancy.

The primary aim of this cohort study was to address existing research gaps by examining the smoking abstinence rate among pregnant individuals who used e-cigarettes compared with NRT in a US national sample. Our secondary aim was to explore the role of timing of e-cigarette initiation by stratifying e-cigarette users into new and existing e-cigarette users, and then comparing each subgroup with NRT users.

## Methods

### Study Population and Sample

This study follows the Strengthening the Reporting of Observational Studies in Epidemiology (STROBE) reporting guideline for cohort studies, and was determined by the University at Buffalo institutional review board to be nonhuman participant research because it was a secondary data analysis of deidentified data, and therefore did not require informed consent in accordance with 45 CFR § 46. Our secondary data analysis was conducted within phase 8 of the US Pregnancy Risk Assessment Monitoring System (PRAMS) between 2016 and 2020.^28^ PRAMS is an ongoing state-level, population-based, surveillance system first administered in 1987 that applies a mixed-mode approach of mail and telephone surveys to collect information on maternal behaviors, attitudes, and experiences before, during, and shortly after pregnancy. Approximately 83% of all US births are covered by PRAMS, including 47 states; Washington, DC; New York, New York; Puerto Rico; and the Great Plains Tribal Chairman’s Health Board.^28^ Written or verbal informed consent was obtained from each participant in the PRAMS study. The deidentified data were provided by the Centers for Disease Control and Prevention, which approved the PRAMS methods and protocol in conjunction with local institutional review boards.^28^

As shown in the Figure, the total sample size was 206 080 individuals in phase 8 of PRAMS. For the purposes of this study, we applied several inclusion criteria to obtain our final analytic sample. First, we narrowed the sample down to the 35 543 individuals who reported smoking cigarettes during the 3 months before pregnancy. Among them, 35 390 had complete data on smoking status during the last 3 months of pregnancy. Because data on NRT use during pregnancy were required for our study, we limited our analysis to 5552 eligible individuals who were residents in the 8 states that had collected data on NRT use (Arkansas, Florida, Georgia, Iowa, Utah, Virginia, Vermont, and West Virginia). Finally, we excluded individuals who (1) used neither NRT nor e-cigarettes (3666 individuals), (2) used both NRT and e-cigarettes (177 individuals), or (3) had missing data on NRT and/or e-cigarette use (380 individuals). Of note, the timing of NRT initiation was not available in the PRAMS questionnaire because the survey only questioned marijuana use during pregnancy.

**Figure.**
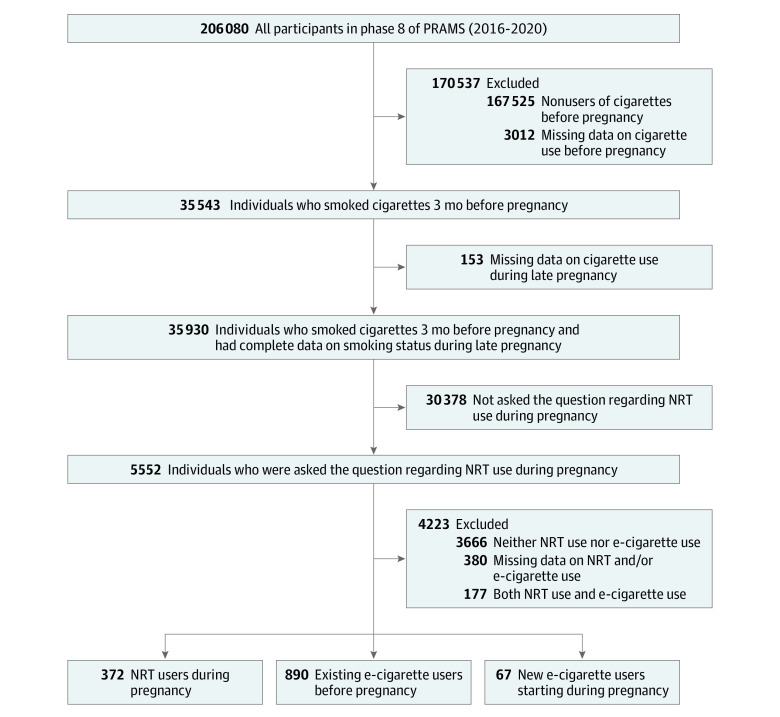
Sample Flowchart E-Cigarette indicates electronic cigarette; NRT, nicotine replacement therapy; PRAMS, Pregnancy Risk Assessment Monitoring System.

To assess the potential for selection bias, we compared sociodemographic characteristics, pregnancy-related characteristics, and substance use characteristics among the 8 participating states that collected the information on smoking cessation methods (eg, NRT use) and the other states participating in the PRAMS study that did not collect this information. Sociodemographic characteristics included age, education level, household income, and race and ethnicity. Race and ethnicity were determined by self-report and included Hispanic or American Indian, non-Hispanic African American, non-Hispanic White, and other race (defined as Asian, Alaska Native, Hawaiian, or multiple races).

### Exposure Measures

In the PRAMS questionnaire, individuals were asked the question, “During your most recent pregnancy, did any of the following things about quitting smoking apply to you*?*” A response option to this question was, “Use a nicotine patch, gum, lozenge, nasal spray or inhaler.” A yes response to this option was defined as the use of NRT during pregnancy. Individuals also reported their e-cigarette use before pregnancy on the basis of the question, “During the 3 months before you got pregnant, on average, how often did you use e-cigarettes or other electronic nicotine products?” The response options included (1) “More than once a day,” (2) “Once a day,” (3) “2 to 6 days a week,” (4) “1 day a week or less,” and (5) “I did not use e-cigarettes or other nicotine-containing e-vaping products then.” A response of 1, 2, 3, or 4 was defined as e-cigarette use, whereas a response of 5 was defined as the nonuse of e-cigarettes before pregnancy. A similar question was asked about e-cigarette use during late pregnancy (“During the last 3 months of your pregnancy, on average, how often did you use e-cigarettes or other electronic nicotine products?”), which had similar response options as e-cigarette use before pregnancy.

In the primary analysis, we compared 2 mutually exclusive groups of individuals who reported cigarette use during pregnancy along with different statuses of NRT and e-cigarette use: NRT users and e-cigarette users. NRT users were defined as individuals who used NRT products during pregnancy. E-Cigarette users were defined as individuals who used e-cigarette products during pregnancy. In the secondary analysis, e-cigarette users were further divided into 2 subgroups (existing e-cigarette users and new e-cigarette users) on the basis of their timing of initiating e-cigarette use before vs during pregnancy. Existing e-cigarette users were defined as individuals who used e-cigarettes before and during pregnancy. New e-cigarette users were defined as individuals who used e-cigarettes during pregnancy but not before pregnancy.

### Outcome Measures

Individuals reported their cigarette use before pregnancy on the basis of the question, “In the 3 months before you got pregnant, how many cigarettes did you smoke on an average day?” Response choices included: (1) 41 cigarettes or more, (2) 21 to 40 cigarettes, (3) 11 to 20 cigarettes, (4) 6 to 10 cigarettes, (5) 1 to 5 cigarettes, (6) less than 1 cigarette, and (7) “I didn’t smoke then.” A response of 1, 2, 3, 4, 5, or 6 was defined as cigarette use, and was used to select the eligible sample for this analysis (ie, cigarette users before pregnancy).

Individuals also reported their cigarette use during late pregnancy using a similar question, “In the last 3 months of your pregnancy, how many cigarettes did you smoke on an average day?” The response options were the same as the response options to the question on cigarette use before pregnancy. Among cigarette users before pregnancy, a response of 7 to the question on cigarette use during late pregnancy was defined as smoking abstinence, and a response of 1, 2, 3, 4, 5, or 6 was defined as continuous smoking.

### Confounders

On the basis of the literature,^29,30,31,32^ we considered the following factors as potential confounders: maternal age (≤19, 20-24, 25-29, and ≥30 years), education (high school diploma or lower, associate’s degree or some college, and bachelor’s degree or higher), type of health insurance (Medicaid, private insurance, self-pay, or other), prepregnancy body mass index (calculated as weight in kilograms divided by height in meters squared), parity (0, 1-2, or ≥3 previous live births), prepregnancy smoking intensity (<1, 1-5, 6-10, 11-20, 21-40, or ≥41 cigarettes per day), depression before pregnancy (yes or no), use of behavioral support for smoking cessation (yes or no), and hookah use (yes or no). Race and ethnicity were not included in the regression due to a substantial amount of missing data on this variable (17.5%), which differed by use of nicotine products.

### Statistical Analysis

Results were reported as weighted percentages and analyses included χ^2^ tests, analysis of covariance, and standardized mean differences, which were used to compare characteristics between NRT users and e-cigarette users (all e-cigarette users, new users, and existing users). To examine the association of smoking cessation aids with smoking abstinence, we first compared the rate of smoking abstinence in late pregnancy between NRT users and e-cigarette users (all users in the primary analysis and new and existing users in the secondary analysis). In the primary analysis, logistic regression models were used to estimate the odds ratio (OR) of smoking abstinence and 95% CI for all e-cigarette users compared with NRT users. In the secondary analysis, we obtained the ORs and the 95% CIs of smoking abstinence for new or existing e-cigarette users compared with NRT users to assess the potential change in effect size by the timing of e-cigarette initiation. Given the substantial differences in baseline characteristics across the groups, we applied propensity score analysis to reduce confounding bias. Propensity score analysis is favorable for cohort studies because it provides a robust estimate of the exposure-outcome association by thoroughly controlling for confounders through a propensity score that represents the probability of occurrence of exposure conditional on observed baseline characteristics (ie, confounders).^33^ Specifically, we created a unique propensity score for each comparison on the basis of essential baseline characteristics that differed between the 2 groups. The NRT group was the reference group for all analyses. We selected confounders on the basis of the literature and results from the univariate analysis in our study. After being created, the propensity score was then included in the regression model as a covariate. Known as a covariate or covariance adjustment, this method of using the propensity score was proposed by Rosenbaum et al^34^ and has been used in various medical research studies.^35,36,37,38,39^

Sampling weights of PRAMS were used in the statistical analysis to reduce potential selection bias due to nonrandom sampling, noncoverage, and nonresponse.^28^ All analyses were conducted using SAS statistical software, version 9.4 (SAS Institute), and a 2-sided *P* ≤ .05 was considered as statistically significant. Data analysis was conducted from March 2022 to April 2023.

## Results

### Sample Characteristics

Our total study sample included 1329 individuals (759 aged ≥25 years [60.2%], 766 non-Hispanic White individuals [79.8%]), which included 890 existing e-cigarette users (unweighted percentage, 67.0%), 67 new e-cigarette users (unweighted percentage, 5.0%), and 372 NRT users (unweighted percentage, 28.0%). The percentage of missing data on race and ethnicity differed by use of nicotine products: 91 NRT users (26.0%), 15 new e-cigarette users (24.2%), and 108 existing e-cigarette users (13.4%). Within the total study sample, a majority had an education level of high school or lower (781 individuals [61.4%]) and had an annual household income of $48 000 or less (952 individuals [81.5%]) (Table 1). Sociodemographic comparisons between the 8 participating states that collected information on smoking cessation methods and the other PRAMS participating states were fairly comparable with a few exceptions. There were greater percentages of individuals aged 29 years or younger, individuals who were non-Hispanic African American, individuals with an education level of high school or lower, and with a low-middle household income in the 8 states (eTable in Supplement 1).

**Table 1.** Sociodemographic, Pregnancy-Related, and Substance Use Characteristics of the Analytic Sample, Stratified by NRT and e-Cigarette Use During Pregnancy, Data From the US PRAMS 2016-2020

Characteristic	Participants, No (%)					*P* value for pairwise comparisons^a^			Standardized mean difference^b^		
	Total sample (N = 1329)	NRT users (n = 372)	E-Cigarette users								
			All users (n = 957)	New users (n = 67)	Existing users (n = 890)	All e-cigarette users vs NRT users	New e-cigarette users vs NRT users	Existing e-cigarette users vs NRT users	All e-cigarette users vs NRT users	New e-cigarette users vs NRT users	Existing e-cigarette users vs NRT users
Age, y											
≤19	106 (7.9)	15 (5.0)	91 (8.6)	1 (0.3)	90 (9.2)	.006	.11	.005	0.14	0.30	0.16
20-24	356 (31.9)	70 (17.3)	286 (35.5)	14 (37.6)	272 (35.3)				0.42	0.47	0.42
25-29	383 (29.7)	115 (37.8)	268 (27.7)	20 (31.3)	248 (27.5)				0.22	0.14	0.22
≥30	376 (30.5)	150 (39.9)	226 (28.2)	27 (30.8)	199 (28.0)				0.25	0.19	0.25
Race and ethnicity											
Hispanic or American Indian	79 (8.2)	12 (5.7)	67 (8.8)	0	67 (9.5)	.67	.01	.64	0.12	0.35	0.14
Non-Hispanic African American	125 (7.2)	49 (10.7)	76 (6.4)	2 (0.5)	74 (6.9)				0.15	0.45	0.13
Non-Hispanic White	766 (79.8)	192 (79.5)	574 (79.9)	45 (99.5)	529 (78.4)				0.01	0.69	0.03
Other race^c^	37 (4.7)	6 (4.1)	31 (4.9)	0	31 (5.3)				0.04	0.29	0.05
Education level											
High school diploma or lower	781 (61.4)	216 (66.1)	565 (60.2)	38 (72.5)	527 (59.3)	.22	.15	.19	0.12	0.14	0.14
Associate’s degree or some college	360 (31.1)	115 (23.6)	245 (32.9)	20 (23.9)	225 (33.6)				0.21	0.01	0.22
Bachelor’s degree or higher	80 (7.6)	19 (10.4)	61 (6.9)	4 (3.6)	57 (7.1)				0.12	0.27	0.11
Household annual income, $											
≤24 000	721 (58.9)	200 (51.8)	521 (60.4)	38 (56.7)	483 (60.7)	.59	.12	.63	0.17	0.10	0.18
24 001-48 000	231 (22.6)	58 (23.4)	173 (22.5)	17 (35.1)	156 (21.5)				0.02	0.26	0.05
48 001-85 000	116 (12.4)	32 (15.4)	84 (11.8)	4 (8.2)	80 (12.1)				0.11	0.23	0.10
≥85 001	54 (6.1)	15 (9.3)	39 (5.4)	0	39 (5.8)				0.15	0.45	0.14
Prenatal care visits, No.											
≤8	324 (24.8)	94 (25.3)	230 (24.6)	22 (30.8)	208 (24.2)	.51	.29	.53	0.02	0.12	0.03
9-11	359 (31.4)	98 (36.9)	261 (30.1)	18 (22.9)	243 (30.7)				0.14	0.31	0.13
≥12	514 (43.8)	150 (37.8)	364 (45.2)	21 (46.3)	343 (45.2)				0.15	0.17	0.15
Parity											
0	500 (44.2)	106 (34.1)	394 (46.7)	21 (34.2)	373 (47.6)	.15	.01	.09	0.26	0	0.28
1-2	541 (40.5)	174 (44.3)	367 (39.6)	25 (16.4)	342 (41.3)				0.10	0.64	0.06
≥3	180 (15.3)	70 (21.5)	110 (13.8)	16 (49.4)	94 (11.1)				0.20	0.61	0.29
Prepregnancy body mass index, mean (SE)^d^	27.2 (0.4)	26.9 (0.8)	27.3 (0.5)	26.2 (1.1)	27.4 (0.5)	.66	.60	.60	0.03	0.06	0.04
Prepregnancy body mass index category^e^											
Underweight	96 (6.0)	24 (5.9)	72 (6.0)	3 (3.2)	69 (6.2)	.99	.60	.99	0.01	0.13	0.02
Normal weight	508 (39.9)	151 (39.5)	357 (39.9)	36 (52.1)	321 (39.0)				0.01	0.25	0.01
Overweight	278 (24.1)	79 (23.2)	199 (24.3)	11 (18.5)	188 (24.7)				0.03	0.11	0.04
Obese	339 (30.1)	96 (31.4)	243 (29.7)	12 (26.2)	231 (30.0)				0.04	0.11	0.03
Gestational hypertension											
Yes	162 (9.9)	48 (11.9)	114 (9.4)	4 (13.2)	110 (9.2)	.54	.91	.50	0.08	0.04	0.09
No	1056 (90.1)	301 (88.1)	755 (90.6)	56 (86.8)	699 (90.8)				0.08	0.04	0.09
Gestational diabetes											
Yes	61 (4.6)	23 (6.6)	38 (4.1)	2 (1.3)	36 (4.3)	.36	.06	.41	0.11	0.27	0.10
No	1157 (95.4)	326 (93.4)	831 (95.9)	58 (98.7)	773 (95.7)				0.11	0.27	0.10
Infant sex											
Male	651 (57.0)	188 (55.8)	463 (57.3)	38 (72.4)	425 (56.1)	.83	.26	.97	0.03	0.35	0.01
Female	570 (43.0)	162 (44.2)	408 (42.7)	24 (27.6)	384 (43.9)				0.03	0.35	0.01
No. of cigarettes smoked/d											
≥41	29 (1.4)	4 (0.4)	25 (1.6)	2 (4.5)	23 (1.4)	<.001	.53	<.001	0.12	0.26	0.10
21-40	119 (7.2)	39 (8.5)	80 (6.9)	8 (3.4)	72 (7.2)				0.06	0.22	0.05
11-20	383 (31.8)	159 (53.1)	224 (26.6)	27 (42.6)	197 (25.4)				0.56	0.21	0.59
6-10	337 (26.9)	91 (20.8)	246 (28.4)	19 (41.9)	227 (27.4)				0.18	0.47	0.15
1-5	275 (23.4)	54 (17.0)	221 (25.0)	6 (7.7)	215 (26.3)				0.20	0.29	0.23
<1	78 (9.3)	3 (0.1)	75 (11.5)	0	75 (12.3)				0.50	0.05	0.52
Behavioral support for smoking cessation											
No	970 (83.9)	200 (65.8)	770 (88.3)	48 (72.1)	722 (89.6)	<0.001	.67	<.001	0.56	0.14	0.60
Yes	251 (16.1)	150 (34.2)	101 (11.7)	14 (27.9)	87 (10.4)				0.56	0.14	0.60
Depression											
No	660 (53.3)	195 (50.4)	465 (54.0)	29 (47.3)	436 (54.5)	.60	.84	.55	0.07	0.06	0.08
Yes	561 (46.7)	155 (49.6)	406 (46.0)	33 (52.7)	373 (45.5)				0.07	0.06	0.08
Hookah use											
No	1056 (80.8)	343 (92.4)	713 (77.9)	60 (96.9)	653 (76.5)	.003	.33	.001	0.42	0.20	0.45
Yes	165 (19.2)	7 (7.6)	158 (22.1)	2 (3.1)	156 (23.5)				0.42	0.20	0.45
Type of health insurance											
Medicaid	844 (64.5)	262 (67.8)	582 (63.7)	45 (75.0)	537 (62.9)	.51	.36	.44	0.09	0.16	0.10
Private insurance, self-pay, or other	363 (35.5)	85 (32.2)	278 (36.3)	14 (25.0)	264 (37.1)				0.09	0.16	0.10

Abbreviations: E-Cigarette, electronic cigarette; NRT, nicotine replacement therapy; PRAMS, Pregnancy Risk Assessment Monitoring System.

^a^ *P* values were calculated using Wald χ^2^ tests.

^b^ Standardized mean difference between 2 means = (μ_1_ − μ_2_) / √[(σ_1_ × σ_1_ + σ_2_ × σ_2_) / 2]; standardized mean difference between 2 proportions = (*P*_1 _− *P*_2_) / √{[*P*_1_ × (1 − P_1_) + *P*_2_ × (1 − *P*_2_)] / 2}.

^c^ Other race was defined as Asian, Alaska Native, Hawaiian, or multiple races.

^d^ Body mass index was calculated as weight in kilograms divided by height in meters squared.

^e^ Body mass index category definitions include underweight (<18.5), normal weight (18.5–24.9), overweight (25.0–29.9), and obese (≥30.0).

A higher percentage of e-cigarette users (286 users [35.5%]) were aged 20 to 24 years compared with the NRT users (70 users [17.3%]) (Table 1). E-Cigarette users were more likely to be moderate cigarette smokers (246 e-cigarette users [28.4%] smoked 6-10 cigarettes per day), whereas NRT users were more likely to be heavy smokers (202 NRT users [62.0%] smoked ≥11 cigarettes per day). Moreover, a lower percentage of e-cigarette users (101 users [11.7%]) received behavioral support than NRT users (150 users [34.2%]). E-Cigarette users (158 users [22.1%]) were more likely to use hookah than NRT users (7 users [7.6%]). These variations were also supported by the standardized mean differences between groups. Other characteristics were comparable between e-cigarette users and NRT users.

Stratified by the timing of initiation, there was a higher percentage of individuals aged 24 years or younger among existing e-cigarette users (362 users [44.5%]) compared with NRT users (85 users [22.3%]). A higher percentage of new e-cigarette users (45 users [99.5%]) were non-Hispanic White, compared with NRT users (192 users [79.5%]) and existing e-cigarette users (529 users [78.4%]). Additionally, a higher percentage of new e-cigarette users (16 users [49.4%]) had 3 or more previous live births, compared with existing e-cigarette users (94 users [11.1%]). For smoking intensity before pregnancy, new e-cigarette users tended to be moderate smokers (19 new e-cigarette users [41.9%] smoked 6 to 10 cigarettes per day), existing e-cigarette users tended to be lighter smokers (290 existing e-cigarette users [38.6%] smoked ≤5 cigarettes per day), and NRT users tended to be heavier smokers (202 NRT users [62.0%] smoked ≥11 cigarettes per day). A lower percentage of existing e-cigarette users (87 users [10.4%]) received behavioral support for smoking cessation than NRT users (150 users [34.2%]). New e-cigarette users (2 users [3.1%]) and NRT users (7 users [7.6%]) were less likely to use hookah than existing e-cigarette users (156 users [23.5%]). Other characteristics were comparable across the 3 groups.

### Rate of Smoking Abstinence in Late Pregnancy by Smoking Cessation Method

The primary analysis revealed that the rate of smoking abstinence in late pregnancy was higher among all e-cigarette users (456 of 957 users [50.8%]) than NRT users (67 of 372 users [19.4%]) (Table 2). The unadjusted OR of smoking abstinence for all e-cigarette users was 3.81 (95% CI, 2.06-7.07; *P* < .001) compared with NRT users. After adjustment of confounders using the propensity score, this association was attenuated, and the adjusted OR was 2.47 (95% CI, 1.17-5.20; *P* = .02).

**Table 2.** Rate of Smoking Abstinence in Late Pregnancy by Smoking Cessation Method Among Cigarette Smokers Before Pregnancy, Data From the US PRAMS, 2016-2020

Smoking cessation method	Participants, No.	Rate of smoking abstinence in late pregnancy					
		Participants, No. (%)	Weighted %	Weighted OR (95% CI)	*P* value	Weighted propensity score–adjusted OR (95% CI)^a^	*P* value
Nicotine replacement therapy	372	67 (18.0)	19.4	1 [Reference]	NA	1 [Reference]	NA
E-Cigarette use							
All	957	456 (47.6)	50.8	3.81 (2.06-7.07)	<.001	2.47 (1.17-5.20)	.02
New e-cigarette use	67	10 (14.9)	20.6	1.08 (0.28-4.25)	.91	1.13 (0.22-5.87)	.88
Existing e-cigarette use	890	446 (50.1)	53.1	4.15 (2.23-7.72)	<.001	2.61 (1.23-5.51)	.01

Abbreviations: E-Cigarette, electronic cigarette; NA, not applicable; OR, odds ratio; PRAMS, Pregnancy Risk Assessment Monitoring System.

^a^ The propensity score was based on the combined information on maternal age, education, prepregnancy body mass index (calculated as weight in kilograms divided by height in meters squared), parity, prepregnancy smoking intensity, depression before pregnancy, use of behavioral support, and hookah use.

Results of the secondary analysis showed that when stratifying e-cigarette users into 2 subgroups of new and existing e-cigarette users, new e-cigarette users (10 of 67 users [20.6%]) had a similar rate of smoking abstinence in late pregnancy compared with NRT users (67 of 372 users [19.4%]) (Table 2). The existing e-cigarette users before pregnancy had a higher rate of smoking abstinence in late pregnancy (446 of 890 users [53.1%]) compared with NRT users (67 of 372 users [19.4%]). The unadjusted OR of smoking abstinence for existing e-cigarette users was 4.15 (95% CI, 2.23-7.72; *P* < .001), compared with NRT users. The propensity score–adjusted OR was attenuated to 2.61 (95% CI, 1.23-5.51; *P* = .01). In contrast, the unadjusted OR of smoking abstinence for new e-cigarette users was 1.08 (95% CI, 0.28-4.25; *P* = .91) compared with NRT users. After adjusting for the propensity score, this association did not change substantially (adjusted OR, 1.13; 95% CI, 0.22-5.87; *P* = .88).

### Supplemental Analyses

Our own supplemental analysis of a PRAMS subsample from the only participating state (Hawaii) that included a question on postpartum e-cigarette use showed that, among cigarette quitters who used e-cigarettes during pregnancy, approximately 50% (1 out of 2 quitters) continued using e-cigarettes in the postpartum period. Additionally, in our sensitivity analysis, the smoking cessation rate among the cohort of 3666 individuals who did not use NRT or e-cigarettes during pregnancy was close to that among the existing e-cigarette users (1759 users [57.9%] vs 446 users [53.1%]). We did not include these nonusers of nicotine products in our main analysis due to concerns for the enormous unmeasured confounders such as cessation motivation and self-efficacy.^40^

Another analysis was done to address the small sample size, especially for the new e-cigarette users (67 users). We conducted a post hoc power analysis using the available sample size and the observed percentages of smoking abstinence. The estimated power (1 − β) was 1.00 for the comparison of all e-cigarette users with NRT users and 0.99 for the comparison of existing e-cigarette users with NRT users, suggesting sufficient statistical power for these 2 comparisons. However, the estimated power was low (0.069) for the comparison between new e-cigarette users and NRT users.

## Discussion

This cohort study of smoking abstinence rates associated with e-cigarette and NRT use among US pregnant smokers identified that individuals who used e-cigarettes during pregnancy had a higher rate of smoking cessation in late pregnancy than individuals who used NRT. Our findings suggested that vaping has the potential to help pregnant individuals quit smoking. Given the substantial perinatal risks associated with tobacco use, ACOG urges clinicians to regularly advise and support pregnant individuals to stop smoking at prenatal and postpartum follow-up visits. It currently recommends psychosocial and behavioral approaches (eg, counseling, support, feedback, and incentives) to quitting smoking during pregnancy.^3^ Neither NRT nor e-cigarette use have been recommended for smoking cessation during pregnancy due to the lack of evidence regarding the efficacy and safety of these products for both the pregnant individual and child.^41^ Our novel findings could help to fill some of these research gaps.

Our observed smoking abstinence rate (19.4%) in late pregnancy among NRT users was comparable with the pooled estimate from a meta-analysis^42^ of 7 studies of pregnant smokers (13.6%). However, the smoking abstinence rate among new e-cigarette users during pregnancy in our sample (20.6%) seemed to be higher than that reported in a 2022 RCT^23^ from the UK (15.6%). This variance might be due to differences in baseline smoking intensity, e-cigarette products, and e-cigarette use adherence. Notably, participants in the UK trial^23^ used e-cigarettes with a legal maximum nicotine concentration of 20 mg/mL (range, 11-20 mg/mL). In contrast, there is no maximum limit for nicotine levels in the US, allowing e-cigarette manufacturers to create products with higher concentrations of nicotine, which may impact both dependency and satisfaction derived from using e-cigarettes.^25,43,44^ Nicotine is most rapidly absorbed via inhalation due to the large surface area of the alveoli and terminal bronchioles of the lungs,^45,46^ which allows the smokers who switched to e-cigarettes to titrate the nicotine level to achieve desired effects quickly. In comparison, absorption of nicotine from NRT is more gradual, leading to a lower likelihood of nicotine dependence.^47^ Additionally, nicotine is metabolized more rapidly during pregnancy due to enhanced activity of the CYP2A6 enzyme while pregnancy-related increases in blood volume further dilutes nicotine levels.^48^ Lower nicotine levels may lead to more nicotine withdrawal symptoms, making cessation more challenging.^29^

Our finding of equivalent outcomes between new e-cigarette users and NRT users might offer more options for pregnant smokers to achieve smoking abstinence. In 2021, a meta-analysis^49^ of 5 RCTs in nonpregnant smokers concluded no significant difference in smoking abstinence rates between e-cigarette and NRT users (rate ratio, 1.42; 95% CI, 0.97-2.09). A 2021 RCT^50^ not included in this meta-analysis^49^ also showed similar smoking cessation rates between the e-cigarette use group and the NRT group (18% vs 20%; risk ratio, 0.91; 95% CI, 0.27-3.03). If smokers prefer not to pursue NRT or have negative experiences and low adherence to NRT use, e-cigarettes could be offered as an alternative smoking cessation aid after a discussion of the potential harms and benefits of using e-cigarettes during pregnancy compared with NRT or continued use of cigarettes.^23,30,31,51^

In addition, our research provides a unique contribution to this field in observations related to the importance of the timing of the initiation of e-cigarettes. Specifically, we found that existing e-cigarette users before pregnancy had a higher smoking abstinence rate in late pregnancy than NRT users. In contrast, new e-cigarette users had a similar smoking abstinence rate to NRT users. Although the factors underlying this difference remain unknown, we propose several possible explanations. First, the unique features of e-cigarettes could make them more attractive to cigarette users than NRT. For example, e-cigarettes support continued smoking-related physical motions (eg, finger-mouth movement) during vaping, produce similar satisfaction as achieved with smoking, and maintain social behaviors and interactions with other smokers.^52^ Second, in contrast with short-term use of NRT,^53^ e-cigarettes may be continued after the target quit date for smoking.^54^ A meta-analysis^55^ of studies on long-term use of e-cigarettes as an aid to smoking cessation found that 54% of participants assigned to e-cigarette conditions continued using e-cigarettes at 6 months or longer after randomization, and 70% of participants who had quit cigarettes were still using e-cigarettes at 6 months or longer after randomization. Third, it may take cigarette users some time to adjust to differences in e-cigarette use, such as deeper inhalation and longer puff duration compared with smoking.^43,56^ Fourth, some existing users who started e-cigarettes prior to pregnancy (dual users) might have already begun transitioning away from traditional cigarette smoking to vaping and, thus, were more likely to achieve smoking abstinence during late pregnancy. This explanation was supported by the substantial reduction in the OR for existing e-cigarette users after adjusting for prepregnancy smoking intensity along with other confounders. Finally, the difference in smoking cessation rate between existing e-cigarette users and NRT users could also be related to sociodemographic and substance use characteristics of existing e-cigarette users including younger age and lighter smoking intensity.

### Limitations

Our study had a few important limitations. First, the small sample size, especially for the new e-cigarette users (67 users), might have produced unreliable results with larger confidence intervals. Second, only 8 of 47 PRAMS participating states collected information on NRT use during pregnancy, which might limit the generalizability of our results to a broader population. However, similar to other states, there is considerable variation among these 8 states in smoke-free air laws (smoking and vaping), as well as access to smoking cessation services including medications (eg, NRT) and counseling.^57^ Third, the PRAMS data we used were collected between 2016 and 2020 and would not capture more recent changes in e-cigarette products, tobacco control policies, and cessation practices. Fourth, most PRAMS data were obtained through retrospective self-reports in the postpartum period, which was subject to recall biases including underreported substance use. Additionally, the questions on e-cigarette use did not explicitly ask whether e-cigarettes were used to aid smoking cessation. Fifth, the information on timing or duration of NRT use was not collected in PRAMS, which did not allow us to stratify NRT users into existing and new users as for e-cigarettes. Sixth, some other potential confounders were not accounted for in our analyses, such as history of smoking cessation, safety perceptions of e-cigarettes and NRT, and advice from health professionals.^30,31,51^ Seventh, except for hookah use, we could not control for other tobacco or nicotine product use (eg, cigars, cigarillos, chewing tobacco, nicotine pouches, and heated tobacco) or other substance use (eg, marijuana, illicit drugs, alcohol, and caffeine) due to lack of detailed information.

It is also important to note that our identified associations cannot be considered causal, especially given substantial confounding by several baseline characteristics, which was supported by the substantial difference between the unadjusted and adjusted ORs for existing e-cigarette users. For example, within our sample, NRT users tended to be heavy smokers, new e-cigarette users tended to be moderate smokers, and existing e-cigarette users tended to be light smokers. In addition, a higher percentage of NRT users and new e-cigarette users received behavioral support compared with existing e-cigarette users. Caution is needed to interpret our propensity score–adjusted associations because this covariate adjustment method may have produced biased estimates, especially if the linearity assumption was not valid.^58,59^ It should be noted that the small sample size and the use of sampling weights of PRAMS prevented us from applying more rigorous methods for the propensity score, such as matching and inverse probability of treatment weighting.^58^

## Conclusions

In summary, our results suggest e-cigarette users overall had a higher smoking abstinence rate during late pregnancy than NRT users. Stratified by the timing of initiation, e-cigarette use before pregnancy was associated with a higher smoking abstinence rate compared with NRT use, but new e-cigarette users during pregnancy had a smoking abstinence rate similar to that of NRT users. These results from our observational research need to be interpreted cautiously and be confirmed in future RCTs to overcome the limitations of lack of data from most recent years (2021-2023), the incomplete exposure measures (especially NRT), and residual confounding effects. Ultimately, e-cigarette products, if shown to have a lower health risk than combustible cigarette use during pregnancy, may support smoking cessation among pregnant individuals and lead to positive changes in clinical recommendations and public health policy.

## Notes

Supplement 1. **eTable.** Sociodemographic, Pregnancy, and Substance Use Characteristics of Study Participants Between the Excluded PRAMS Sample and the Analytic Subsample of 8 States With Data on Smoking Cessation Methods

Supplement 2.
Data Sharing Statement
