# Error in Methods and Figure

**DOI:** 10.1001/jamanetworkopen.2023.38725

**Published:** 2023-10-05

In the Original Investigation titled “Cigarette Smoking Abstinence Among Pregnant Individuals Using E-Cigarettes or Nicotine Replacement Therapy,”^1^ published September 12, 2023, there were errors in the Figure (35 930 should have been 35 390, and 30 378 should have been 29 838). In the Study Population and Sample subsection of the Methods, the word “marijuana” should have been “NRT” in this sentence: “Of note, the timing of NRT initiation was not available in the PRAMS questionnaire because the survey only questioned marijuana use during pregnancy.” This article has been corrected.^1^
