# Supplementary material for: Cigarette Smoking Abstinence Among Pregnant Individuals Using E-Cigarettes or Nicotine Replacement Therapy
Source: JAMA Netw Open. 2023 Sep 12;6(9):e2330249. doi: 10.1001/jamanetworkopen.2023.30249 (PMC10498331; doi:10.1001/jamanetworkopen.2023.30249)
Supplement: Supplement 1. — eTable. Sociodemographic, Pregnancy, and Substance Use Characteristics of Study Participants Between the Excluded PRAMS Sample and the Analytic Subsample of 8 States With Data on Smoking Cessation Methods [file jamanetwopen-e2330249-s001.pdf]

# Supplemental Online Content

Wen X, Chung MV, Liszewski KA, et al. Cigarette smoking abstinence among pregnant individuals using e-cigarettes or nicotine replacement therapy. *JAMA Netw Open.* 2023;6(9):e2330249. doi:10.1001/jamanetworkopen.2023.30249

**eTable.** Sociodemographic, Pregnancy, and Substance Use Characteristics of Study Participants Between the Excluded PRAMS Sample and the Analytic Subsample of 8 States With Data on Smoking Cessation Methods

This supplemental material has been provided by the authors to give readers additional information about their work.

© 2023 Wen X et al. *JAMA Netw Open.*

## eTable. Sociodemographic, Pregnancy, and Substance Use Characteristics of Study Participants Between the Excluded PRAMS Sample and the Analytic Subsample of 8 States With Data on Smoking Cessation Methods

| Characteristic | Excluded sample of states *without* data on smoking cessation methods (N=173,476) % | Analytic sub-sample of 8 states *with* data on smoking cessation methods (N=32,604)* % | P-value** |
|---|---|---|---|
| **Age, years** | | | **<0.001** |
| ≤19 | 4.3 | 4.6 | |
| 20-24 | 18.1 | 20.0 | |
| 25-29 | 28.8 | 29.8 | |
| ≥30 | 48.9 | 45.5 | |
| **Race/ethnicity** | | | **<0.001** |
| Non-Hispanic Caucasian | 56.8 | 55.7 | |
| Non-Hispanic African American | 14.2 | 19.5 | |
| Hispanic or American Indian | 20.0 | 18.4 | |
| Other race | 9.0 | 6.5 | |
| **Education level** | | | **<0.001** |
| High school or lower | 36.4 | 39.1 | |
| Associate's degree or some college | 26.7 | 26.8 | |
| Bachelor's degree or higher | 36.8 | 34.2 | |
| **Household annual income** | | | **<0.001** |
| ≤$24000 | 32.7 | 32.0 | |
| $24,001-$48,000 | 19.3 | 22.0 | |
| $48,001-$85,000 | 18.9 | 20.8 | |
| ≥$85,001 | 29.1 | 25.2 | |
| **Method of payment** | | | **<0.001** |
| Medicaid | 41.8 | 39.5 | |
| Private Insurance | 52.0 | 51.7 | |
| Self-pay | 3.0 | 4.7 | |
| Other | 3.2 | 4.1 | |
| **Number of prenatal care visits** | | | 0.068 |
| ≤8 | 18.4 | 17.8 | |
| 9 to 11 | 31.1 | 30.4 | |
| ≥12 | 50.6 | 51.8 | |
| **Number of live births** | | | **<0.001** |
| 0 | 38.8 | 38.9 | |
| 1-2 | 49.7 | 48.3 | |
| ≥3 | 11.5 | 12.8 | |
| **Pre-pregnancy BMI, kg/m$^2$** | | | 0.055 |
| Underweight | 3.5 | 3.4 | |
| Normal weight | 45.0 | 44.1 | |
| Overweight | 26.1 | 25.6 | |

© 2023 Wen X et al. *JAMA Netw Open.*

| Characteristic | Excluded sample of states *without* data on smoking cessation methods (N=173,476) % | Analytic sub-sample of 8 states *with* data on smoking cessation methods (N=32,604)* % | P-value** |
|---|---|---|---|
| Obese | 25.4 | 26.8 | |
| **Gestational hypertension** | | | **0.003** |
| Yes | 9.8 | 8.9 | |
| No | 90.2 | 91.1 | |
| **Gestational diabetes** | | | **0.033** |
| Yes | 7.1 | 6.5 | |
| No | 92.9 | 93.5 | |
| **Infant sex** | | | **0.032** |
| Male | 50.8 | 52.0 | |
| Female | 49.2 | 48.0 | |
| **Number of cigarettes smoke** | | | **<0.001** |
| ≥41 | 0.3 | 0.2 | |
| 21 to 40 | 0.9 | 0.9 | |
| 11 to 20 | 4.0 | 3.6 | |
| 6 to 10 | 4.4 | 4.0 | |
| 1 to 5 | 5.1 | 4.3 | |
| <1 | 1.7 | 1.3 | |
| 0 | 83.5 | 85.8 | |
| **Depression** | | | 0.784 |
| Yes | 85.8 | 85.9 | |
| No | 14.2 | 14.1 | |
| **Hookah Use** | | | 0.164 |
| Yes | 95.4 | 95.1 | |
| No | 4.6 | 4.9 | |
| **Type of health insurance** | | | **<0.001** |
| Medicaid | 41.8 | 39.5 | |
| Private insurance/self-pay/other | 58.2 | 60.5 | |
| **Frequency of e-cigarette use before pregnancy** | | | 0.304 |
| More than once a day | 1.7 | 1.8 | |
| Once a day or 2-6 days a week | 1.0 | 1.1 | |
| 1 day a week or less | 1.7 | 1.6 | |
| Not using electronic vapor products | 95.6 | 95.4 | |

PRAMS, Pregnancy Risk Assessment Monitoring System; BMI, body mass index.
* The 8 participating states included Arkansas, Florida, Georgia, Iowa, Ulta, Virginia, Vermont, and West Virginia.
** P-values from Wald Chi-square tests.

© 2023 Wen X et al. *JAMA Netw Open.*
