# Supplementary material for: Cigarette Smoking Abstinence Among Pregnant Individuals Using E-Cigarettes or Nicotine Replacement Therapy
Source: JAMA Netw Open. 2023 Sep 12;6(9):e2330249. doi: 10.1001/jamanetworkopen.2023.30249 (PMC10498331; doi:10.1001/jamanetworkopen.2023.30249)
Supplement: Supplement 2. — Data Sharing Statement [file jamanetwopen-e2330249-s002.pdf]

# Data Sharing Statement

Wen. Cigarette Smoking Abstinence Among Pregnant Individuals Using e-Cigarettes or Nicotine Replacement Therapy. *JAMA Netw Open*. Published September 12, 2023. doi:10.1001/jamanetworkopen.2023.30249

**Data**
**Data available:** No

**Additional Information**
**Explanation for why data not available:** The Pregnancy Risk Assessment Monitoring System (PRAMS) data used for this study was provided by the Centers for Disease Control and Prevention (CDC). The authors are not allowed to share the data according to the signed External Researcher Data Sharing Agreement with the CDC. Researchers who are interested in using the PRAMS data can submit a request to the CDC staff. More details on access to the PRAM data can be found on the website (https://www.cdc.gov/prams/prams-data/researchers.htm).
